# Determination of genetic effects and functional SNPs of bovine *HTR1B* gene on milk fatty acid traits

**DOI:** 10.1186/s12864-021-07893-8

**Published:** 2021-07-27

**Authors:** Mingyue Cao, Lijun Shi, Peng Peng, Bo Han, Lin Liu, Xiaoqing Lv, Zhu Ma, Shengli Zhang, Dongxiao Sun

**Affiliations:** 1 Department of Animal Genetics, Breeding and Reproduction, College of Animal Science and Technology, Key Laboratory of Animal Genetics, Breeding and Reproduction of Ministry of Agriculture and Rural Affairs, National Engineering Laboratory for Animal Breeding, China Agricultural University, Beijing, 100193 China; 2 Institute of Animal Science, Chinese Academy of Agricultural Sciences, Beijing, 100193 China; 3 Beijing Dairy Cattle Center, Beijing, 100192 China

**Keywords:** Genetic effects, *HTR1B*, Milk fatty acids, Dairy cattle, Luciferase assay

## Abstract

**Background:**

Our previous genome-wide association study (GWAS) on milk fatty acid traits in Chinese Holstein cows revealed, the SNP, BTB-01556197, was significantly associated with C10:0 at genome-wide level (*P* = 0.0239). It was located in the down-stream of 5-hydroxytryptamine receptor 1B (*HTR1B*) gene that has been shown to play an important role in the regulation of fatty acid oxidation. Hence, we considered it as a promising candidate gene for milk fatty acids in dairy cattle. In this study, we aimed to investigate whether the *HTR1B* gene had significant genetic effects on milk fatty acid traits.

**Results:**

We re-sequenced the entire coding region and 3000 bp of 5′ and 3′ flanking regions of *HTR1B* gene. A total of 13 SNPs was identified, containing one in 5′ flanking region, two in 5′ untranslated region (UTR), two in exon 1, five in 3′ UTR, and three in 3′ flanking region. By performing genotype-phenotype association analysis with SAS9.2 software, we observed that 13 SNPs were significantly associated with medium-chain saturated fatty acids such as C6:0, C8:0 and C10:0 (*P* < 0.0001 ~ 0.042). With Haploview 4.1 software, linkage disequilibrium (LD) analysis was performed. Two haplotype blocks formed by two and ten SNPs were observed. Haplotype-based association analysis indicated that both haplotype blocks were strongly associated with C6:0, C8:0 and C10:0 as well (*P* < 0.0001 ~ 0.0071). With regards to the missense mutation in exon 1 (g.17303383G > T) that reduced amino acid change from alanine to serine, we predicted that it altered the secondary structure of HTR1B protein with SOPMA. In addition, we predicted that three SNPs in promoter region, g.17307103A > T, g.17305206 T > G and g.17303761C > T, altered the binding sites of transcription factors (TFs) HMX2, PAX2, FOXP1ES, MIZ1, CUX2, DREAM, and PPAR-RXR by Genomatix. Of them, luciferase assay experiment further confirmed that the allele T of g.17307103A > T significantly increased the transcriptional activity of *HTR1B* gene than allele A (*P* = 0.0007).

**Conclusions:**

In conclusion, our findings provided first evidence that the *HTR1B* gene had significant genetic effects on milk fatty acids in dairy cattle.

**Supplementary Information:**

The online version contains supplementary material available at 10.1186/s12864-021-07893-8.

## Background

Milk fat, a vital nutritional ingredient of milk, is considered as one of the economic traits of milk production in dairy cattle [1]. Triglyceride synthesized by fatty acids and α-glycerophosphate in mammary epithelial cells is the main component of milk fat [2]. Fatty acids contain saturated fatty acids (SFAs) and unsaturated fatty acids (UFAs). In SFAs, C12:0, C14:0, C16:0 increases low-density lipoprotein cholesterol and risk of cardiovascular diseases [3], while C15:0, C17:0 are inversely associated with cardiometabolic risk [4]. UFAs are beneficial for reducing the risk of heart and other diseases [5, 6]. Many previous studies have shown that the phenotypic variation of milk fatty acid compositions were genetically controlled and the heritability estimates were around 0.14 ~ 0.33 for SFAs and 0.08 ~ 0.29 for UFAs in Holstein cattle [7–11].

One of genome-wide significant SNPs, BTB-01556197 associated with C10:0 (*P* = 0.0239) identified in previous GWAS [12], was located in the down-stream of 5-hydroxytryptamine receptor 1B (*HTR1B*) gene. The bovine *HTR1B* gene has merely one exon spanning 4305 bp and was involved in the c-AMP signaling pathway that was related to PI3K-Akt pathway, a well-known pathway for fat synthesis and metabolism [13]. Hence, we considered the *HTR1B* gene as a promising candidate for milk fatty acid traits in dairy cattle. In the present study, we aimed to further preform association analysis in a different Chinese Holstein population to confirm the genetic effects of *HTR1B* on milk fatty acids and to identify potential functional genetic variations.

## Results

### SNPs identification

A total of 13 SNPs (Table 1) was identified, including one (g.17307103A > T) in 5′ flanking region, two (g.17305206 T > G and g.17303761C > T) in 5′ untranslated region (UTR), two (g.17303383G > T and g.17303042C > G) in the exon 1, five (g.17302291C > G, g.17302078G > T, g.17301689 T > C, g.17301647G > T and g.17299803A > G) in 3′ UTR, and three (g.17298882A > G, g.17296725C > T and g.17296695 T > C) in 3′ flanking region. Of note, g.17303383G > T in exon 1 was a missense mutation resulting in an amino acid replacement from alanine (GCC) to serine (TCC). The detailed information, and genotypic and allele frequencies of the 13 SNPs were shown in Table 1.

**Table 1:** Detailed information of 13 SNPs identified in *HTR1B* gene

SNP name	Location	Position (UMD 3.1.1)	EVA no.	Genotype	NO.	Frequency	Allele	Frequency
g.17307103A > T	5′ flanking region	Chr9: 17307103	rs207969357	AA	481	0.4621	A	0.6830
				TT	100	0.0961	T	0.3170
				TA	460	0.4419		
g.17305206 T > G	5′ UTR	Chr9: 17305206	rs476055046	TT	651	0.6242	T	0.8121
				GG	0	0.0000	G	0.1879
				GT	392	0.3758		
g.17303761C > T	5′ UTR	Chr9: 17303761	rs133683693	CC	113	0.1088	C	0.3503
				TT	424	0.4081	T	0.6497
				CT	502	0.4832		
g.17303383G > T	Exon-1	Chr9: 17303383	rs209984404	GG	504	0.4846	G	0.6986
				TT	91	0.0875	T	0.3014
				GT	445	0.4279		
g.17303042C > G	Exon-1	Chr9: 17303042	rs208945882	CC	499	0.4897	C	0.6977
				GG	96	0.0942	G	0.3023
				CG	424	0.4161		
g.17302291C > G	3′ UTR	Chr9: 17302291	rs136136524	CC	279	0.2680	C	0.5259
				GG	225	0.2161	G	0.4741
				CG	537	0.5159		
g.17302078G > T	3′ UTR	Chr9: 17302078	rs135063494	GG	502	0.4818	G	0.6972
				TT	91	0.0873	T	0.3028
				GT	449	0.4309		
g.17301689 T > C	3′ UTR	Chr9: 17301689	rs379078023	CC	103	0.1012	C	0.3089
				TT	492	0.4833	T	0.6911
				CT	423	0.4155		
g.17301647G > T	3′ UTR	Chr9: 17301647	rs109548495	GG	115	0.1103	G	0.3514
				TT	425	0.4075	T	0.6486
				GT	503	0.4823		
g.17299803A > G	3′ UTR	Chr9: 17299803	rs208790360	AA	499	0.4854	A	0.6989
				GG	90	0.0875	G	0.3011
				AG	439	0.4270		
g.17298882A > G	3′ flanking region	Chr9: 17298882	rs380460000	AA	2	0.0038	A	0.5019
				GG	0	0.0000	G	0.4981
				AG	523	0.9962		
g.17296725C > T	3′ flanking region	Chr9: 17296725	rs208087947	CC	503	0.4846	C	0.6985
				TT	91	0.0877	T	0.3015
				CT	444	0.4277		
g.17296695 T > C	3′ flanking region	Chr9: 17296695	rs133617481	CC	90	0.0872	C	0.2994
				TT	504	0.4884	T	0.7006
				CT	438	0.4244		

Note: *EVA* European Variation Archive, *UTR* untranslated region

### Associations between the SNPs with 23 milk fatty acids traits

By performing association analysis on the SNPs with 23 kinds of milk fatty acids with SAS9.2, significant genetic associations were observed (Additional file 1: Table S1). The SNP, g.17307103A > T, was significantly associated with C6:0, C8:0, C10:0, C16:1, C16 index and C17 index (*P* = 0.0003 ~ 0.042). Both g.17305206 T > G and g.17303761C > T were significantly associated with C6:0, C10:0, C12:0, C14:0, C18:1cis-9, C18 index, SFA, UFA and SFA/UFA (*P* < 0.0001 ~ 0.036), and they were significantly associated with three (C8:0, C16:1 and C20:0; *P* < 0.0001 ~ 0.0213) and two (C17:0, *P* = 0.0358; and C17 index, *P* = 0.0148) milk fatty acid traits, respectively. Six SNPs were significantly associated with seven milk fatty acid traits, namely, g.17303383G > T (C6:0, C8:0, C10:0, C14:0, C16:1, C16 index, and C17 index; *P* = 0.0003 ~ 0.0145), g.17303042C > G (C8:0, C10:0, C14:0, C16:1, C17:0, C16 index, and C17 index; *P* < 0.0001 ~ 0.0286), g.17302291C > G (C6:0, C8:0, C10:0, C17:0, SFA and SFA/UFA; *P* = 0.0172 ~ 0.0387), g.17302078G > T (C6:0, C8:0, C10:0, C14:0, C16:1, C16 index and C17 index; *P* < 0.0001 ~ 0.0277), g.17299803A > G (C6:0, C8:0, C10:0, C14:0, C16:1, C16 index and C17 index; *P* = 0.001 ~ 0.0305), and g.17296725C > T (C6:0, C8:0, C10:0, C14:0, C16:1, C16 index and C17 index; *P* < 0.0001 ~ 0.0122). The g.17301689 T > C showed strong associations with C6:0, C8:0, C10:0, 16:1, C16 index and C17 index (*P* = 0.0002 ~ 0.0204). The g.17301647G > T, showed strong associations with C6:0, C10:0, C12:0, C14:0, C17:0, C18:1cis-9, C17 index, SFA, UFA and SFA/UFA (*P* < 0.0001 ~ 0.0445). The g.17298882A > G was significantly associated with C14:0, C17:0, C17:1, C18:1cis-9, C20:0, SFA, UFA and SFA/UFA (*P* < 0.0001 ~ 0.049). The g.17296695 T > C was significantly associated with C6:0, C8:0, C10:0, C14:0, C16:1, C17:0, C16 index and C17 index (*P* < 0.0001 ~ 0.0492). While no significant association was observed for C11:0, C13:0, C14:1, C15:0, C16:0, C18:0 and C14 index (*P* > 0.05). After multiple-testing, eight SNPs were still significantly associated with five milk fatty acid traits, namely, g.17305206 T > G (C6:0, C8:0, C10:0,UFA; *P* < 0.0001), g.17303761C > T (C6:0; *P* < 0.0001), g.17303042C > G (C8:0; *P* < 0.0001), g.17302078G > T (C8:0; *P* < 0.0001), g.17301647G > T (C6:0; *P* < 0.0001), g.17298882A > G (C20:0; *P* < 0.0001), g.17296725C > T (C8:0; *P* < 0.0001) and g.17296695 T > C (C8:0; *P* < 0.0001).

Correspondingly, the additive (*a*), dominance (*d*), and allele substitution (*α*) effects of the identified SNPs were calculated (Additional file 2: Table S2), and the results showed that 11 SNPs had strong genetic effects on C6:0, C8:0, C10:0, C12:0, C14:0, C16:1, C17:0, C18:0, C18:1cis-9, C18 index, C20:0, C16 index, C17 index, SFA, UFA and SFA/UFA (*P* < 0.05).

### Associations between the haplotype blocks with 23 milk fatty acid traits

**Fig. 1:**
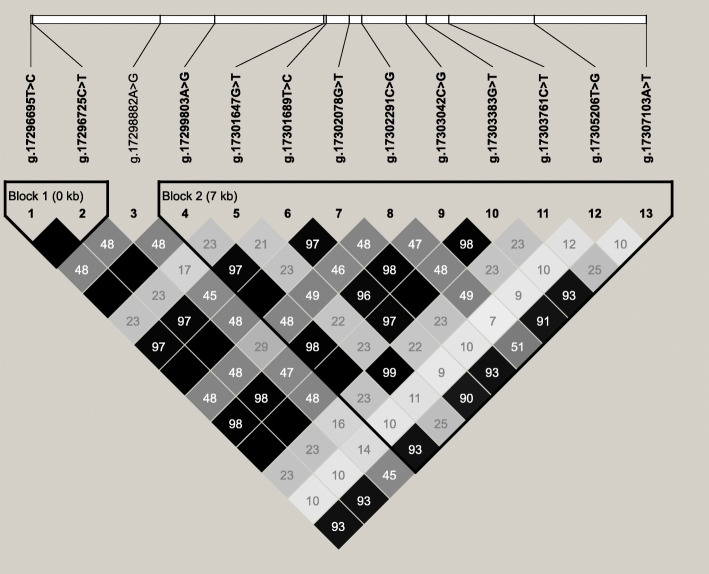
Linkage disequilibrium (LD) among the 13 SNPs of *HTR1B* gene

Among the 13 SNPs identified in this study, two haplotype blocks were observed with the Haploview 4.1 software (Fig. 1). The block 1, formed by g.17296725C > T and g.17296695 T > C, had two kinds of haplotypes (H1 = TC and H2 = CT) with the frequencies of 69.6 and 30.4%, respectively. The block 2 was composed of ten SNPs, g.17307103A > T, g.17305206 T > G, g.17303761C > T, g.17303383G > T, g.17303042C > G, g.17302291C > G, g.17302078G > T, g.17301689 T > C, g.17301647G > T and g.17299803A > G, and had seven haplotypes (H1 = AGTGCCGCTA, 34.5%; H2 = GTCTGGTTTT, 30.3%; H3 = ATTGGCGTGA, 13.8%; H4 = ATTGCCGTTA, 12.9%; H5 = ATTGCCGTGA, 4.4%; H6 = ATTGGCGTTA, 1.6%; and H7 = ATTGGCGTTT, 1.5%).

Subsequently, we performed haplotype-based association analysis with SAS9.2, and found that the haplotype block1 and block2 were significantly associated with nine (C6:0, C8:0, C10:0, C14:0, C16:1, 17:0, C20:0, C16 index and C17 index; *P* = 0.0002 ~ 0.0476) and five (C6:0, C8:0, C10:0, C20:0 and C17 index; *P* < 0.0001 ~ 0.0265) milk fatty acids, respectively (Additional file 3: Table S3). While, none of significant associations were detected with C11:0, C12:0, C13:0, C14:1, C15:0, C16:0, 17:1, C18:0, C18:1cis-9, C18 index, C14 index, SFA, UFA and SFA/UFA (*P* > 0.05). Through multiple testing, the haplotype block1 and block2 were still significantly associated with one (C8:0; *P* = 0.0002) and three (C6:0, C8:0, C10:0; *P* < 0.0001) milk fatty acids, respectively.

### Changes of the HTR1B protein secondary structure and function caused by the missense mutation g.17303383G > T

Using SOPMA software, we predicted that the missense mutation in exon 1, g.17303383G > T, changed the HTR1B protein secondary structure, including α-helix (46.90 to 41.44%), extended strand (13.40 to 16.38%), β-turn (2.48 to 1.74%), and random coil (37.22 to 40.45%) with the allele from G to T. While, the HTR1B protein function was not altered by the missense mutation with the scores 0.25 (SIFT) and − 1.42 (PROVEAN).

### Changes of transcriptional activity caused by g.17307103A > T, g.17305206 T > G and g.17303761C > T

By searching the TFBSs of the two SNPs in 5′ UTR and one SNP in 5′ flanking region with Genomatix, we discovered that the allele T of g.17307103A > T created two TFBSs for HMX2 (Hmx2/Nkx5–2 homeodomain transcription factor) and PAX2 (Zebrafish PAX2 paired domain protein), and the allele G of g.17305206 T > G created a TFBS for FOXP1ES (Alternative splicing variant of FOXP1, activated in ESCs). For g.17303761C > T, the allele C created two TFBSs for MIZ1 (Myc-interacting Zn finger protein 1, zinc finger and BTB domain containing 17) and CUX2 (Cut-like homeobox 2, dimeric binding site), and the allele T created two TFBSs for DREAM (Downstream regulatory element-antagonist modulator, Ca2 + −binding protein of the neuronal calcium sensors family that binds DRE sites as a tetramer) and PPAR-RXR (PPAR/RXR heterodimers, DR1 sites). The detailed results were shown in Table 2.

**Table 2:** Changes of transcription factor binding sites (TFBSs) caused by the SNPs in the 5′ UTR and flanking region of *HTR1B*

SNP	Sequence	TF	Full name
g.17307103A > T	CCCCAACGCGATTCCCTCCTT		
	CCCCAACGCGTTTCCCTCCTT	HMX2	Hmx2/Nkx5–2 homeodomain transcription factor
		PAX2	Zebrafish PAX2 paired domain protein
g.17305206 T > G	TTTTGAAGTTTTTTTTTTTTTT		
	TTTTGAAGTTTGTTTTTTTTTT	FOXP1ES	Alternative splicing variant of FOXP1, activated in ESCs
g.17303761C > T	ACCTCGCCCTCGACCTCTCGC	MIZ1	Myc-interacting Zn finger protein 1, zinc finger and BTB domain containing 17 (ZBTB17)
		CUX2	Cut-like homeobox 2, dimeric binding site
	ACCTCGCCCTTGACCTCTCGC	DREAM	Downstream regulatory element-antagonist modulator, Ca2 + −binding protein of the neuronal calcium sensors family that binds DRE (downstream regulatory element) sites as a tetramer
		PPAR-RXR	PPAR/RXR heterodimers, DR1 sites

Notes: *TF* transcrition factor

**Fig. 2:**
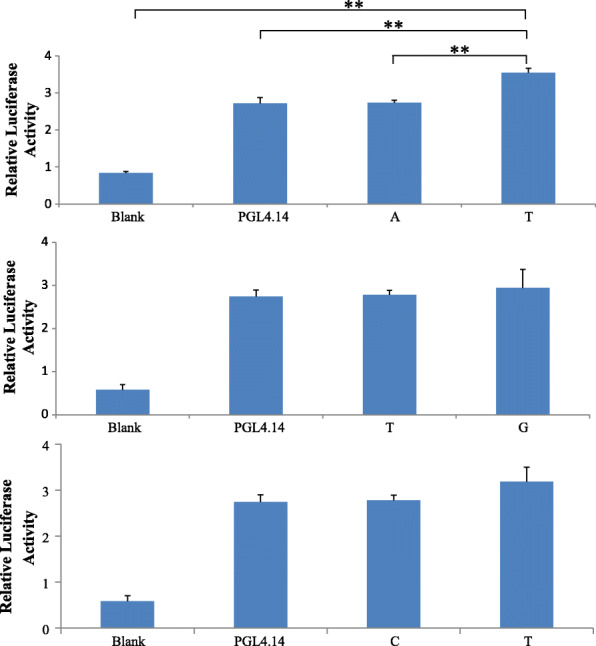
Luciferase assay analysis of the recombinant plasmids in HEK293 cells. Blank: Blank cells. PGL4.14: Empty vector. A and T: Plasmids of g.17307103A > T. T and G: Plasmids of g.17305206 T > G. C and T: Plasmids of g.17303761C > T. **: *P* < 0.01

Further, we utilized the luciferase assay (Fig. 2) to confirm the above prediction results. The luciferase activities of the construct T of g.17307103A > T was observed significantly higher than those of the blank control (*P* < 0.0001), the empty vector PGL4.14 (*P* = 0.0022), and the construct A (*P* = 0.0007), indicating that the allele T of g.17307103A > T increased the transcriptional activity of *HTR1B* compared with allele A. However, the luciferase activities of four constructs (T and G of g.17305206 T > G, and C and T of g.17303761C > T) were not strongly changed than those of the blank and empty vector, implying that g.17305206 T > G and g.17303761C > T did not significantly alter the transcriptional activity of *HTR1B* gene.

## Discussion

According to our previous GWAS results and biological functions, the *HTR1B* gene has been identified as one of novel promising candidates for milk fatty acids in dairy cattle [12]. In the present study, we firstly confirmed that the *HTR1B* gene showed significant genetic effects on medium-chain saturated fatty acids in dairy cattle, providing a basis for further verification.

Previous studies reported that protein secondary structure could be used to build safe starting cores to generate the complete protein fold [14], and to set structural constraints for protein threading [15, 16]. Studies reported that the missense mutations caused by the sequence variations were related to the protein function to account for the phenotype variations [17–20]. Here, we identified a missense mutation (g.17303383G > T), and it changed the protein secondary structure by prediction with the SOPMA. While we used SIFT and PROVEAN softwares to detect that the missense mutation did not alter the HTR1B protein function. Hence, the significant associations of g.17303383G > T with milk fatty acids might be due to the LD with the true causal mutations.

Regulatory region, including promoter, enhancer, silencer, and insulator etc. [21], are important for the gene regulation and expression [22]. Transcription factors (TFs) are the sequence-specific DNA-binding proteins that regulate gene expression in all organisms [23, 24], and approximately 10% are implicated in diverse diseases in human [25]. In eukaryotes, multiple TFs cooperatively bind regulatory DNA to temporally and spatially control gene expression [26]. Generally speaking, 5’ UTR plays regulatory roles in the gene expression through binding transcription factors or unknown regulatory mechanisms. Hence, we wish to investigate if the SNPs in the 5’ UTRs changed the expression of *HTR1B* gene. It has been well-known that transcription factors (TFs) play an important role in gene expression and transcriptional regulation [27, 28]. Previous studies found that both 5’ UTR and 5’ flanking region can combine with TF, and some SNPs within these regions may change the TF-binding thereby leading to changes in gene transcription activity and eventually affecting related traits [29–31]. In this study, we predicted that three SNPs (g.17307103A > T, g.17305206 T > G and g.17303761C > T) changed the TFBSs. While only g.17307103A > T actually changed the transcriptional activity of *HTR1B* gene. The allele T of g.17307103A > T was predicted to create two TFBSs for HMX2 and PAX2. The *hmx* homeobox-containing TF gene family, containing contains *hmx2* [32], is highly conserved across species [33–36]. HMX2 is involved in a feedback loop of EGF signaling and located in upstream of the *PAX5* of the utricular maculae to affect the inner ear development in zebrafish [37, 38]. The *pax* gene family encodes DNA binding TFs that control vital steps in embryonic development and differentiation of specific cell lineages in human [39]. PAX2 as a TF can promote the expression of *ADAM10* to negatively regulate the epithelia to mesenchyme transition in human [40]. According to the significant associations and transcriptional activity caused by g.17307103A > T, we suggested that g.17307103A > T might be a potential causal mutation regulating the *HTR1B* gene expression by altering the binding sits for the TFs HMX2 and PAX2 to impact the milk fatty acid traits in dairy cattle. Further investigation is needed to validate the regulatory of specific transcription factors.

Regarding the expression of the *HTR1B* gene in multiple tissues, based on the RNA-seq database, Cattle Gene Atlas (http://cattlegeneatlas.roslin.ed.ac.uk/) [41], we observed that the *HTR1B* gene was expressed in 82 tissues/ cell types, including mammary gland, while *HTR1B* gene was moderately expressed in mammary gland.

## Conclusions

In this study, we confirmed the significant genetic effects of the *HTR1B* gene on milk fatty acids using post-GWAS strategy, and identified a potential functional mutation in 5′-flanking region, g.17307103A > T, that altered the transcriptional activity of *HTR1B*. Our findings provided valuable molecular information for genetic improvement programs in dairy cattle.

## Methods

### Animals and phenotypic data

A total of 1065 Chinese Holstein cows was used in this study that were different from those in our previous GWAS [12]. They were from 44 sire families with an average of 24 daughters per sire and collected in the 23 dairy farms of the Beijing Sanyuanlvhe Dairy Farming Center (Beijing, China). We collected milk samples of these 1065 cows to measure milk fatty acids in the laboratory of Beijing Dairy Cattle Center (www.bdcc.com.cn). By gas chromatograph, the contents of 16 kinds of main milk fatty acids were directly detected, including SFA: C6:0, C8:0, C10:0, C11:0, C12:0, C13:0, C14:0, C15:0, C16:0, C17:0, C18:0, C20:0; and UFA: C14:1, C16:1, C17:1 and C18:1cis-9. Based on these phenotype values, we obtained four index traits (C14 index, C16 index, C17 index and C18 index) calculated with the formulas: $\frac{\mathrm{cis}-9\ \mathrm{unsaturated}}{\mathrm{cis}-9\ \mathrm{unsaturated}+\mathrm{saturated}}\ast 100$ [42], and summarized the SFA, UFA, SFA/UFA.

### Gas chromatograph

Total milk fat were extracted from approximately 2 ml of each milk sample. Fatty acids have high boiling points, so they are unstable and easy to crack at high temperatures. The high temperature in gas chromatographic analysis will cause the loss of fatty acids, so pre-treatment is required. Therefore, when analyzing fatty acids and fats, especially fatty acid components, to reduce the boiling point and improve stability, we react the fatty acids or fats with methanol to prepare fatty acid methyl esters and then perform gas chromatography analysis.

The measurement conditions of the gas chromatograph are as follows: the injector temperature is 260 °C; carrier gas (helium) flow rate is 45 mL/min; split ratio is 100:1; chromatographic column conditions: keep at 100 °C for 10 min, heat up at 6 °C /min to 160 °C and hold for 10 min, heat up at 5 °C /min to 200 °C and hold for 20 min, heat up at 4 °C /min to 240 °C and hold for 12 min; the detector temperature is 260 °C.

### SNP identification and genotyping

We extracted genomic DNA from the blood samples of 1065 cows and the semen samples of 44 sires using TIANamp Blood DNA Kit (Tiangen, Beijing, China) and salt-out procedure, respectively. DNA quantity and quality were measured by NanoDrop™ ND-2000 Spectrophotometer (Thermo Scientific, Hudson, DE, USA) and 2.0% agarose gel electrophoresis.

A total of 15 pairs of primers (Additional file 4: Table S4) were designed for PCR amplification using the Primer 3.0 (http://primer3.wi.mit.edu/) based on the sequences of all the exons, and 3000 bp of 5′ and 3′ flanking regions of the bovine *HTR1B* gene (Gene ID: 317707), and were synthesized in the Beijing Genomics Institute (Beijing, China). By using the DNA samples of the abovementioned 44 sires with the same concentration of 50 ng/μl, two DNA pools were constructed and 22 sires were included in each pool. Then, PCR amplification was performed with abovementioned 15 pairs of primers and PCR procedure was as follows: initial denaturation at 94 °C for 5 min; annealing at 94 °C for 30 s, 60 °C for 30 s and 72 °C for 30 s, for 35 cycles and final extension at 72 °C for 7 min. By sequencing PCR products, we identified potential polymorphic sites.

Then, 1065 cows were individually genotyped by using matrix-assisted laser desorption/ionization time of flight mass spectrometry (MALDI-TOF MS, Sequenom MassARRAY, Bioyong Technologies Inc. HK). As for each identified SNP, PCR amplification was first performed with sequence-specific extension primers, then 1 base was extended targeting two alleles of the identified SNP. According to different mass-to-charge ratios of two alleles, different mass spectrum peaks could be observed to detect the genotype of each SNP.

### Statistical analysis

First, we used the Haploview 4.1 software (Broad Institute of MIT and Harvard, Cambridge, MA, USA) to identify the LD extent among the identified SNPs of the *HTR1B* gene.

Subsequently, we performed single SNP-based and haplotype-based association analysis. We traced the pedigrees of the 1065 Chinese Holstein cows back to three generations, as a result, a total of 3335 individuals were included for association analysis, which kinship matrix (**A**-matrix) were constructed with SAS9.2 (SAS Institute, Cary, NC, USA). Then, associations between the identified SNPs and haplotype blocks with 23 milk fatty acid traits were performed by SAS9.2 on the basis of the following mixed animal model:

$$ {Y}_{ijklm}=\upmu +{G}_i+{h}_j+{l}_k+{a}_l+\mathrm{b}\times {M}_m+{e}_{ijklm} $$

Here, *Y*_*ijklm*_ is the phenotypic value of each milk fatty acid trait; μ is the overall mean; *G*_*i*_ is the fixed effect corresponding to the genotype or haplotype combination; *h*_*j*_ is the fixed effect of farm (*j* = 1 ~ 23); *l*_*k*_ is the fixed effect of stage of lactation (*k* = 1 ~ 4); *a*_*l*_ is the random polygenic effect; *M*_*m*_ is the fixed effect of age at calving (*m* = 1 ~ 293); b is the regression coefficient of covariate M; and *e*_*ijklm*_ is the random residual. Bonferroni correction was performed according to the number of multiple tests, in which the adjusted significance levels of *P* < 0.05 for the single SNP and haplotype-based analysis were 0.0002 and 0.0011, respectively.

Further, we calculated the additive effect (a), dominant effect (d), and substitution effect (α) of SNP on the milk fatty acid traits according to the formulas [43]: $\alpha =\frac{AA- BB}{2},d= AB-\frac{AA+ BB}{2}, and\ \alpha =\alpha +d\left(q-p\right)$, in which, *p* and *q* were the frequencies of A and B, respectively; and AA, AB and BB were the least square means of fatty acids corresponding to the genotypes.

### Prediction of the secondary structure and function changes of the HTR1B protein

We used the NPSA SOPMA SERVER program (https://npsa-prabi.ibcp.fr/cgi-bin/npsa_automat.pl?page=npsa_sopma.html) to predict whether the identified missense mutation in coding region changed HTR1B protein secondary structure, and set the parameters with similarity threshold (8), and number of states (4-Helix, Sheet, Turn, Coil). Also, we used the SIFT (http://sift.bii.a-star.edu.sg/) and PROVEAN (http://sift.jcvi.org/index.php) to investigate whether the missense mutation altered the protein function. The score thresholds of the SIFT and PROVEAN were 0.05 [44] and − 2.5 [45], respectively. When the score is below the threshold, the protein function is considered changed.

### Prediction of the changes of transcription factor binding sites (TFBSs)

We predicted whether the SNPs in 5′ UTR and flanking region of the *HTR1B* gene impacted on TFBSs by using the Genomatix suite v3.9 (http://www.genomatix.de/cgi-bin/welcome/welcome.pl?s=d1b5c9a9015b02bb3b1a806f9c03293f).

### Construction of recombinant plasmid, cell culture and luciferase assay

**Fig. 3:**
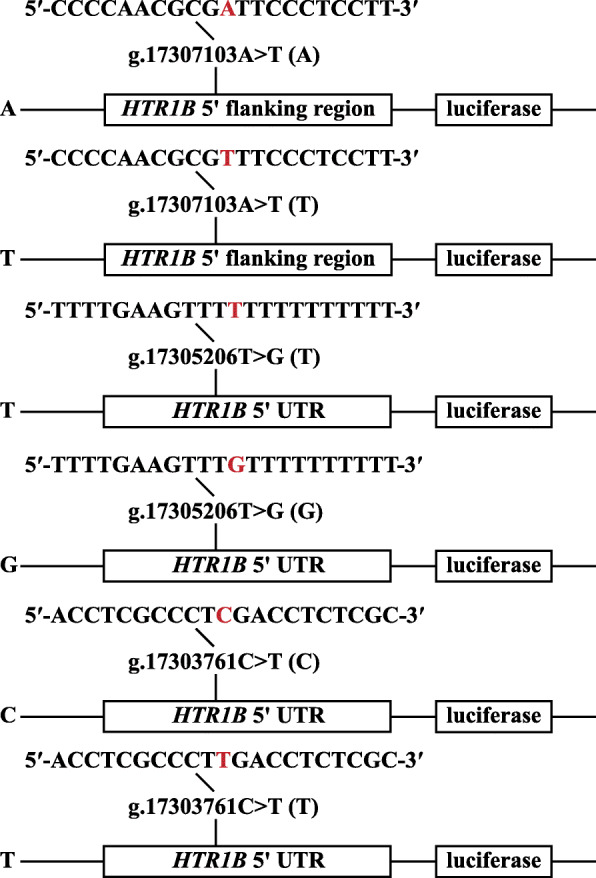
Sketches of recombinant plasmids. The nucleotides in red highlight referred to the SNP

**Fig. 4:**
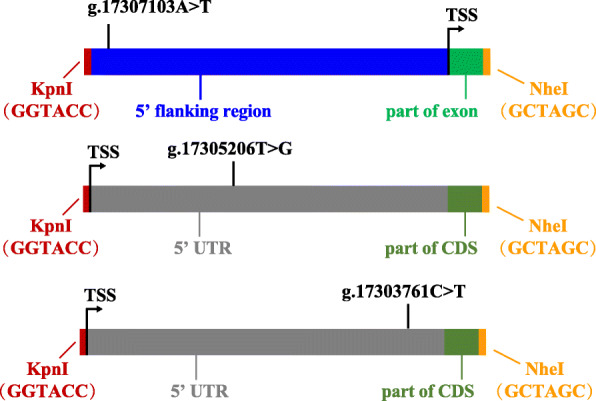
The constructions of the cloned fragments of three SNPs. KpnI (GGTACC) and NheI (GCTAGC) are restriction enzyme cutting sites. TSS: transcription start site. CDS: coding sequence

We constructed six luciferase reporter gene fragments with Kpn1 and Nhel restriction sites at the 5′ to 3′ termini (Figs. 3 and 4), which contained alleles A and T of g.17307103A > T, T and G of g.17305206 T > G, and C and T of g.17303761C > T. The six fragments were synthesized in Genewiz (Suzhou, China), and were cloned into the pGL4.14 Luciferase Assay Vector (Promega, Madison, USA). Subsequently, the plasmids were purified by Endo-free Plasmid DNA Mini Kit II (OMEGA, omega bio-tek, Norcross, Georgia, USA), and then were sequenced to confirm the integrity of each construct’s insertions.

Human Embryonic Kidney (HEK)-293 T cells were cultured in Dulbecco’s modified Eagle’s medium (DMEM) including 10% heat-inactivated fetal bovine serum (FBS; Gibco, Life Technologies) at 5% CO_2_ and 37 °C. We seeded approximately 2 × 10^5^ cells per well into 24-well plates, and transfected the cells using Lipofectamine 2000 (Invitrogen, CA, USA). For each well, we transfected 500 ng of the constructed plasmid DNA along with 10 ng of pRL-TK renilla luciferase reporter vector (Promega), and conducted three replicates for each construct. The cells were cultured for about 36-48 h after transfection and then were measured the activities of firefly and renilla luciferases using a Dual-Luciferase Reporter Assay System (Promega) with a Modulus microplate multimode reader (Turner Biosystems, CA, USA). Finally, average statistics of three replicates were calculated as the normalized luciferase data (firefly/renilla).

## Supplementary Information

**Additional file 1: Table S1**. Associations of 13 SNPs of *HTR1B* gene with fatty acid traits in Chinese Holstein (LSM ± SE).

**Additional file 2: Table S2**. Additive(a), dominant(d) and substitution(α) effects of 11 SNPs on milk fatty acid traits of *HTR1B* gene.

**Additional file 3: Table S3**. Haplotype associations of the haplotype blocks in *HTR1B* gene with milk fatty acids (LSM ± SE).

**Additional file 4: Table S4**. PCR primers information of *HTR1B* gene.

## Data Availability

All data generated or analysed during this study are included in this published article and its supplementary information files.

## Abbreviations

a: Additive effect
CDS: coding sequence
d: Dominant effect
EVA: European Variation Archive
FA: Fatty acids
GWAS: Genome-wide association study
*HTR1B*: 5-hydroxytryptamine receptor 1B
LD: Linkage disequilibrium
PCR: Polymerase chain reaction
SFA: Saturated fatty acids
SNP: Single nucleotide polymorphism
TF: Transcription factor
TFBS: Transcription factor binding site
TSS: transcription start site
UFA: Unsaturated fatty acids
UTR: Untranslated region
α: Substitution effect
